# Antipyretics and vaccine‐induced myocarditis

**DOI:** 10.1002/clc.24026

**Published:** 2023-05-30

**Authors:** Stephen A. Hoption Cann

**Affiliations:** ^1^ School of Population & Public Health, Faculty of Medicine University of British Columbia Vancouver British Columbia Canada

Fatima et al.^1^ discuss potential mechanisms for the development of myocarditis and pericarditis following mRNA vaccination for COVID‐19. The authors note that “myocarditis has been reported as a side effect of other vaccines such as the smallpox vaccine. However, the smallpox vaccine differs from the COVID‐19 vaccine in its composition and induced action.” While this is true, they do have some things in common. One feature they share is that myocarditis is more common following the vaccine dose associated with the greater side effects. For smallpox vaccines in vaccine‐naïve recipients, this occurs after the first dose of ACAM2000 and Dryvax. For mRNA vaccines, this occurs after the second dose. These common side effects include: fever, headache, myalgia, injection site pain, and so forth. More prevalent side effects, in turn, would then lead to higher doses and more frequent use of the other thing that they have in common—antipyretics. The limited number of antipyretics used to treat symptoms from these vaccines (generally either ibuprofen or acetaminophen), narrows down potential suspects.

The challenge is that case reports and case series on vaccine‐associated myocarditis often only discuss symptoms at presentation (i.e., several days or more after vaccination) and thus avoid a discussion of side effects that were directly related to the vaccination itself, and rarely mention whether antipyretics were used or not.^2^ This potential mechanism—not discussed in this review or any other reviews on this topic—needs further investigation.

Meune et al.^3^ reviewed studies examining the effect of various NSAIDs (ibuprofen, aspirin, and indomethacin) on viral myocarditis development in animal models. They noted that studies examining early administration of NSAIDs found these drugs exacerbated the myocarditis and increased myocarditis‐related mortality.^4^, ^5^, ^6^ In contrast, studies examining the effects of NSAIDs in the late phase of viral myocarditis showed no negative effects^7^ or lesser effects than seen relative to early administration.^8^

As these drugs are frequently used in patients who have symptoms such as fever or chest pain, proving any causation by antipyretics in the development of myocarditis is very difficult. While this is a great challenge, it is not insurmountable. Finding the link between aspirin use during viral infections and the subsequent development of Reye syndrome was equally challenging due to its widespread use. Like vaccine‐associated myocarditis, Reye syndrome usually develops 3–5 days after the onset of a viral illness. Shortly following Reye and colleagues description of the syndrome in 1963, salicylates were mentioned as a suspect,^9^ but were often disregarded.^10^, ^11^, ^12^ A key problem, as some authors commented, was that “most children with fever are given aspirin.”^13^ It would end up taking decades before an association was finally accepted.^14^

Now we find ourselves again in a similar situation, where most individuals with vaccine‐associated side effects treat them with an antipyretic, but on the rare occasion where it is actually reported, there is no discussion of a possible role in myocarditis.^15^ Considering that these are drugs used after vaccination, but before the typical period when symptoms of myocarditis develop (usually several days or more following vaccination), they should remain under suspicion until evidence is presented otherwise. While evidence to implicate antipyretics in vaccine‐associated myocarditis does not exist, neither is there evidence to rule them out. Therefore, case–control studies with meticulous gathering of data on antipyretic use are essential to support continued recommendations for their use following vaccination.
